# Gold Nanoparticles Modulate BCG-Induced Innate Immune Memory in Human Monocytes by Shifting the Memory Response towards Tolerance

**DOI:** 10.3390/cells9020284

**Published:** 2020-01-23

**Authors:** Benjamin J. Swartzwelter, Francesco Barbero, Alessandro Verde, Maria Mangini, Marinella Pirozzi, Anna Chiara De Luca, Victor F. Puntes, Luciana C. C. Leite, Paola Italiani, Diana Boraschi

**Affiliations:** 1 Institute of Biochemistry and Cell Biology, CNR, Via P. Castellino 111, 80131 Napoli, Italy; benjamin.swartzwelter@ibbc.cnr.it (B.J.S.); alessandro.verde@ibbc.cnr.it (A.V.); maria.mangini@ibbc.cnr.it (M.M.); marinella.pirozzi@ibbc.cnr.it (M.P.); annachiara.deluca@ibbc.cnr.it (A.C.D.L.); paola.italiani@ibbc.cnr.it (P.I.); 2 Institut Català de Nanociència i Nanotecnologia (ICN2), CSIC and The Barcelona Institute of Science and Technology (BIST), Campus UAB, Bellaterra, 08193 Barcelona, Spain; francesco.barbero@icn2.cat (F.B.); victor.puntes@icn.cat (V.F.P.); 3 Laboratório de Desenvolvimento de Vacinas, Instituto Butantan, São Paulo SP 05503-900, Brazil; luciana.leite@butantan.gov.br

**Keywords:** innate immune memory, gold nanoparticles, monocytes, potentiation, BCG, inflammatory cytokines, anti-inflammatory cytokines

## Abstract

Innate immune memory is characterized by a modulation in the magnitude with which innate immune cells such as monocytes and macrophages respond to potential dangers, subsequent to previous exposure to the same or unrelated agents. In this study, we have examined the capacity of gold nanoparticles (AuNP), which are already in use for therapeutic and diagnostic purposes, to modulate the innate memory induced by bacterial agents. The induction of innate memory was achieved in vitro by exposing human primary monocytes to bacterial agents (lipopolysaccharide -LPS-, or live Bacille Calmette-Guérin -BCG) in the absence or presence of AuNP. After the primary activation, cells were allowed to return to a resting condition, and eventually re-challenged with LPS. The induction of memory was assessed by comparing the response to the LPS challenge of unprimed cells with that of cells primed with bacterial agents and AuNP. The response to LPS was measured as the production of inflammatory (TNFα, IL-6) and anti-inflammatory cytokines (IL-10, IL-1Ra). While ineffective in directly inducing innate memory per se, and unable to influence LPS-induced tolerance memory, AuNP significantly affected the memory response of BCG-primed cells, by inhibiting the secondary response in terms of both inflammatory and anti-inflammatory factor production. The reprogramming of BCG-induced memory towards a tolerance type of reactivity may open promising perspectives for the use of AuNP in immunomodulatory approaches to autoimmune and chronic inflammatory diseases.

## 1. Introduction

Innate immune memory is defined as the ability of innate immune cells to react differently to challenges based on previous stimulations [1,2,3]. While innate immune memory is the only type of immune memory in plants, invertebrates, and lower vertebrates that lack adaptive immunity, innate memory in higher vertebrates co-exists with adaptive immunological memory, which forms the basis of more effective and highly antigen-specific secondary responses [4,5,6]. In mammals, innate memory is non-specific, but in any case, aimed at developing a better-suited secondary response. The best-known phenomenon of innate memory in mammals is the so-called endotoxin tolerance, i.e., the decreased response to gram-negative bacterial endotoxin (lipopolysaccharide, LPS) upon repeated challenges, which aims at limiting the tissue damage that could be caused by a recurrent host inflammatory reaction [7,8,9,10]. More recent evidence has shown that previous challenge with some bacteria, such as the *Mycobacterium bovis* strain BCG, can induce an innate memory towards an enhanced and more protective secondary response (“trained” immunity) [11,12,13,14].

Among the innate immune cells that can develop memory, monocytes and macrophages are particularly important because of their evolutionarily conserved immune memory capacity and their role in modulating local immune responses, in addition to their direct ability to uptake foreign and endogenous agents that may pose a threat [15,16,17,18]. The protective scavenging role of monocytes/macrophages presents the question of whether and how foreign materials can impact innate immune memory in monocytes and macrophages. This is a critical issue that has to be considered in novel treatments and therapies that use biomedical materials. One class of materials that may have a particular impact on innate memory, because of their particulate nature, are nanomaterials, which have extensive clinical relevance and potential as metal-based imaging agents and as drug or vaccine carriers [19,20]. 

A wealth of studies on the immunological safety of medical nanomaterials have been conducted, leading to the design of nanoparticles (NP) that are immunocompatible, i.e., unable to trigger an immune/inflammatory reaction [21,22,23]. Gold NP (AuNP) are one of these immunosafe particles [24] and have already attained clinical relevance for uses such as specific cell targeting in photothermal and radiation-based treatments [25,26]. Beyond these, extensive potential exists for AuNP uses in a variety of diagnostic and therapeutic applications in humans [27]. For a more thorough assessment of the possible impact of AuNP on the host immune competence, it is important to investigate the effects of AuNP on innate memory, i.e., their capacity of altering or modulating the immune defensive reactivity to subsequent infectious or stressful agents/events. Some preliminary data suggest that this may be the case [28]. If indeed it could be possible to modulate innate immune/inflammatory reaction with AuNP, this would open the possibility of targeted interventions for limiting excessive inflammation in autoimmune, chronic inflammatory, and degenerative diseases, and likewise, to increase immune reactivity in situations of age- or disease-caused immunosuppression. 

The aim of the present study is to investigate whether AuNP are capable of inducing innate immune memory in human monocytes, and/or whether they may modulate memory induced by bacterial agents. We have used primary monocytes for studying the development of innate memory, thereby employing an in vitro model that reproduces a repeated exposure to foreign agents. Blood monocytes were chosen, as opposed to resident tissue macrophages, as they are the main inflammatory cells that engage with foreign materials during a tissue reaction and therefore those that most likely can develop memory of previous challenges. The use of human primary cells, rather than transformed cell lines or animals/animal cells, would ensure a higher predictivity and an improved relevance for the human situation in vivo.

The results of this study confirm that AuNP do not activate innate immune/inflammatory reactions, being therefore in principle immunologically “safe”. However, we show here that AuNP can significantly modulate innate responses and innate memory induced by BCG, while remaining inactive the responses and memory induced by LPS. This suggests that AuNP can be used for regulating human innate reactivity and opens the way to future therapeutic applications.

## 2. Materials and Methods

### 2.1. Synthesis and Characterization of AuNP

#### 2.1.1. AuNP Synthesis

Synthesis of AuNP was conducted using wet chemistry methods as previously described by Bastús et al. [29]. Briefly, a 150 mL aqueous solution of sodium citrate (2.2 mM) was brought to a boil under reflux, after which 1 mL of 25 mM HAuCl_4_ was quickly injected into the citrate solution. Within several minutes the solution obtained a red hue, which is indicative of AuNP formation, resulting in ~10 nm NP seeds. This was followed by sequential growth-inducing steps, which consisted of adding further HAuCl_4_ to the sample solution, leading to the desired AuNP size. All reagents were obtained from Sigma-Aldrich, Inc. (St. Louis, MO, USA).

#### 2.1.2. STEM, UV-Vis, DLS, and Z-Potential Characterization

STEM (scanning transmission electron microscopy) images were acquired using a FEI Magellan XHR scanning electron microscope (SEM) (FEI, Hillsboro, OR, USA), operated in transmission mode at 20 kV. AuNP samples stabilized with polyvinylpyrrolidone (55 kDa) to avoid aggregation were drop-cast (4 μL) onto a carbon-coated copper TEM grid and left to dry at room temperature. Images were acquired, and at least 500 particles from different regions of the grid were computer-counted using an in-house ImageJ macro. 

UV-Vis spectra of AuNP suspensions in sodium citrate were acquired using a Shimadzu UV-2400 spectrophotometer (SSI; Kyoto, Japan), reading a spectral range from 300 to 750 nm. Acquisitions occurred at room temperature with AuNP samples placed in 1 mL cuvettes, and milliQ water was used as a reference. 

The hydrodynamic diameter and Z-potential of AuNP in sodium citrate were acquired by dynamic light scattering and laser doppler velocimetry, using a Malvern Zetasizer Nano ZS instrument (Malvern Panalytical Ltd., Malvern, UK) equipped with a light source wavelength of 632.8 nm and a fixed scattering angle of 173°. Diameters were reported as distribution intensity calculated by non-negative least squares (NNLS) analysis. The software was arranged with the parameters of the refractive index and the absorption coefficient of AuNP and the solvent viscosity of water at 25 °C. 

### 2.2. LAL Assay

Endotoxin contamination of AuNP was assessed using the chromogenic Pyrochrome LAL assay (Associates of Cape Cod, Inc.; East Falmouth, MA, USA), following a protocol that has been optimized for use with nanoparticulate samples [30]. Interference of AuNP with the LAL assay readout was assessed, and AuNP were tested at concentrations below the interference threshold. LAL contamination was expressed in terms of endotoxin units per milligram of AuNP (EU/mg). 

### 2.3. Human Monocyte Isolation

Blood was obtained from healthy donors. All subjects gave their informed consent for inclusion before they participated in the study. The study was conducted in accordance with the Declaration of Helsinki, and the protocol was approved by the Regional Ethics Committee for Clinical Experimentation of the Tuscany Region (Ethics Committee Register n. 14,914 of May 16, 2019). None of the subjects was vaccinated with BCG or was positive in the tuberculin test. Peripheral blood mononuclear cells (PBMC) were obtained by Ficoll-Paque gradient density separation (GE Healthcare, Bio-Sciences AB, Uppsala, Sweden). CD14^+^ monocytes were isolated from PBMC using anti-CD14 antibody-bearing magnetic microbeads (Miltenyi Biotec, Bergisch Gladbach, Germany) according to the manufacturer’s instructions. Cell viability was assessed by trypan blue dye exclusion and found to be > 95%. Monocyte purity was determined microscopically after cytocentrifugation and differential staining with a modified Wright-Giemsa dye (Diff-Quik; Medion Diagnostics, Duedingen, Switzerland). Only preparations with >95% purity were used. 

### 2.4. Monocyte Stimulation

#### 2.4.1. Biocorona Formation on AuNP

AuNP were incubated for 1 h at 37 °C in heat-inactivated human AB serum (Sigma-Aldrich, Inc.) at a 1:1 ratio, allowing a protein bio-corona to form around the NP. Aggregation of particles upon addition to cell culture was thus avoided [31]. The AuNP-serum mixture was used directly for stimulation of monocytes, accounting for any serum added concurrently with the AuNP.

#### 2.4.2. Primary Monocyte Response

Freshly isolated monocytes were suspended in culture medium (RPMI 1640 + Glutamax-I; GIBCO by Life Technologies, Paisley, UK) supplemented (unless otherwise noted) with 50 µg/mL gentamicin sulfate (GIBCO), and 1 × 10^5^ cells/well were seeded in 96-well flat bottom plates (Corning^®^ Costar^®^; Corning Inc. Life Sciences, Oneonta, NY, USA). For primary stimulation, monocytes were incubated, in a final volume of 0.2 mL, with culture medium (negative control), AuNP (10 µg/mL), LPS (1 ng/mL; from *E. coli* O55:B5; Sigma-Aldrich, Inc.), live BCG (*Mycobacterium bovis* BCG Moreau, at a multiplicity of infection (MOI) of 1; Instituto Butantan, São Paulo, Brazil), LPS + AuNP, or BCG + AuNP. Final AB serum concentration was adjusted at 5%. Priming in BCG experiments occurred in the absence of antibiotics. The MOI = 1 was selected from dose-response experiments as the lowest dose that yielded full cell activation in the absence of cell death (not shown). The LPS concentration was selected from dose–response experiments as a non-toxic concentration able to induce significant tolerance type memory (data not shown). After 24 h supernatants were collected and frozen at −20 °C for subsequent cytokine analysis. By visual inspection, cell viability did not change in response to the different treatments.

#### 2.4.3. Secondary Monocyte Response

Following primary stimulation, culture wells were replenished with fresh culture medium containing 5% AB serum, and cells were rested for 6 days, refreshing medium every 2 days. The resting period was selected as sufficient for achieving return to baseline production of inflammatory cytokines in cells previously activated for 24 h with LPS (0.1–10 ng/mL) or BCG (0.1–10 MOI). At day 7, cells were challenged with either culture medium or 5 ng/mL LPS (a 5× higher concentration than in the primary response) for 24 h, and then supernatants were collected and frozen at −20 °C for subsequent cytokine analysis. Cell viability did not change in response to the different treatments (daily visual inspection of each well).

### 2.5. Transmission Electron Microscopy

Cells in 6-well plates (1 × 10^6^ cells/well) were exposed to AuNP (20 µg/mL) alone or in the presence of LPS (1 ng/mL) or BCG (MOI = 1) for 24 h. Cells were then fixed in 1% glutaraldehyde diluted in HEPES (0.2 M) for 1 h at room temperature, washed with PBS, placed in PBS + 1% BSA and carefully dislodged using a plastic scraper. Cells were washed and pelleted by centrifugation, stained with osmium tetroxide and uranyl acetate, and progressively dehydrated using 30%, 50%, 70%, 90%, and 100% EtOH, and then incubated in acetone. The cell pellet was embedded in an EPON resin and polymerized by baking at 60 °C for 48 h, and ultrathin sections (70 nm) were obtained using a Leica EM UC7 ultramicrotome (Leica Microsystems). TEM images were obtained using a FEI Tecnai 12 transmission electron microscope.

### 2.6. Cytokine Analysis

The levels of the inflammatory cytokines TNFα and IL-6 and of the anti-inflammatory factors IL-1Ra and IL-10 were assessed by ELISA (R&D Systems, Minneapolis, MN, USA) following the manufacturer’s instructions. Absorbance of assay wavelength was measured at 450 nm (subtracting background present at 550 nm) using a Cytation 3 imaging reader (BioTek, Winooski, VT, USA).

### 2.7. Statistical Analysis

Data from cytokine measurements have been analyzed using GraphPad Prism7 software (GraphPad Inc., La Jolla, CA, USA), and are presented in terms of ng/10^6^ plated monocytes. Results are reported as mean ± SEM of values from different donors/experiments. The use of SEM is meant for showing the precision of the mean. In each experiment, two replicate wells were prepared for each experimental point, and two separate ELISA determinations were run on each well. Statistical significance of differences is indicated by *p* values, calculated using a paired Student’s two tail *t*-test. 

## 3. Results

### 3.1. Effect of AuNP on Innate Memory Responses Induced by LPS 

**Figure 1:**
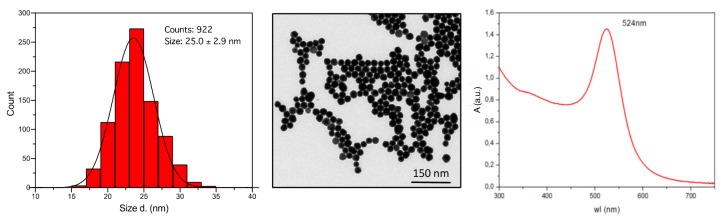
Characterization of 25 nm AuNP. Size distribution profile (**left panel**), STEM image (**central panel**), and UV-Vis profile (**right panel**) of AuNP following synthesis.

In this study, we have used AuNP with an average size of 25 ± 2.9 nm dispersed in 2.2 mM sodium citrate (Figure 1). UV-Vis spectroscopy revealed a maximum absorbance of 524 nm with no additional peaks, indicating that particles were free from aggregation. DLS demonstrated a hydrodynamic diameter of 32.8 ± 10.4 nm and a Z-potential of −31.3 ± 0.6 mV. By modified LAL assay, the endotoxin contamination of the AuNP batch used in this study was 9.36 EU/mg. Pre-incubation in human serum prevented aggregation upon subsequent addition to cell cultures [31].

**Figure 2:**
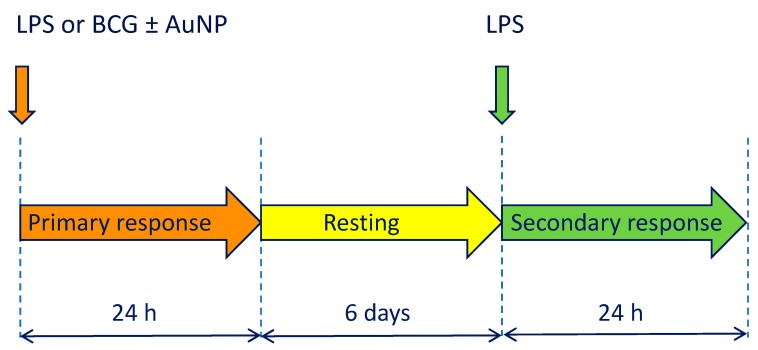
Schematic depiction of the in vitro model of innate immune memory. Primary activation was induced by stimulation for 24 h with LPS or BCG, in the absence or in the presence of AuNP. Supernatants were collected and cells rested for six days. On day 7, a secondary response was induced by challenge with LPS for 24 h (control groups were challenged with medium alone), after which supernatants were collected for evaluation of cytokine production.

We have investigated whether AuNP are capable of inducing innate memory in human monocytes. To this end, we have used primary cells isolated from the blood of healthy donors in an in vitro model of repeated challenges (Figure 2). Primary monocytes were exposed in culture to medium alone, AuNP, the bacterial agents LPS (lipopolysaccharide from E. coli) or BCG (live bacteria), or to the mixture of bacterial agents with AuNP. Monocyte activation was measured in terms of production of inflammation-related factors after 24 h. After a 6-day period of resting in culture, cells were exposed to a secondary challenge (a higher concentration of LPS, i.e., 5 ng/mL) and their response again assessed. The concentration of AuNP selected for these experiments has been chosen as the highest concentration at which the endotoxin contamination was below monocyte activation threshold. The AuNP size was selected from preliminary experiments in which NP of different size were tested (Supplementary Figure S1). Uptake of AuNP was assessed by TEM and found comparable in the different groups by visual inspection (Supplementary Figure S2).

**Figure 3:**
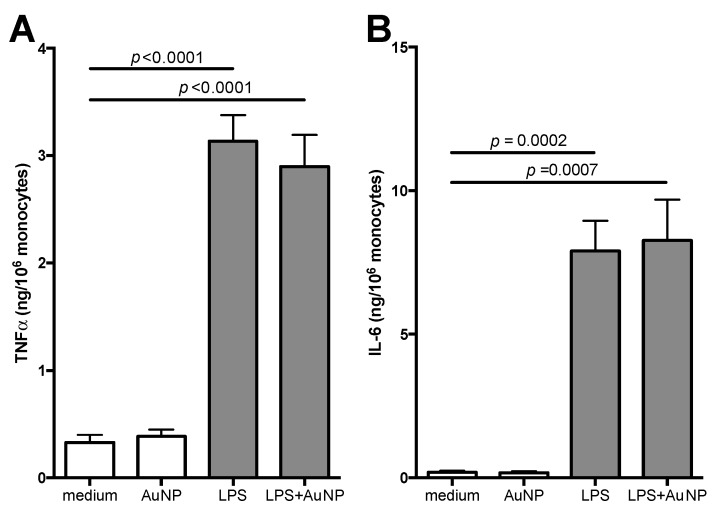
Primary response of human monocytes to LPS and AuNP. CD14^+^ monocytes were exposed for 24 h to LPS (1 ng/mL) in the absence or in the presence of AuNP (10 μg/mL). Release of TNFα (**A**) and IL-6 (**B**) was measured in the supernatants. Results are reported as mean ± SEM of data from 4 individual donors, tested in four separate experiments. *p* values are indicated when *p* < 0.05. Although statistically significant, we have not reported in the figure the differences between AuNP and LPS (*p* < 0.0001 for TNFα, and *p* = 0.0002 for IL-6) or between AuNP and LPS + AuNP (*p* < 0.0001 for TNFα, and *p* = 0.0007 for IL-6).

**Figure 4:**
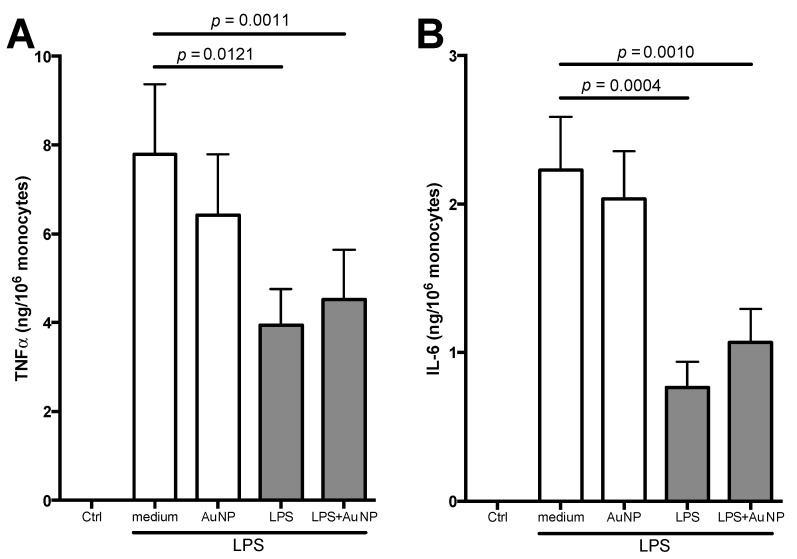
Secondary response of human monocytes primed with LPS and AuNP. Following primary activation (in x-axis: medium, AuNP, LPS, LPS + AuNP), cells were rested for six days and then challenged with LPS (5 ng/mL) for 24 h. Supernatants were tested for TNFα (**A**) and IL-6 (**B**). Ctrl represents the response of cells challenged with medium (the lack of response was identical in cells previously primed with medium, AuNP, LPS, or LPS + AuNP). Results are reported as mean ± SEM of data from 4 individual donors, tested in four separate experiments. Further, *p* values are indicated when *p* < 0.05. Although statistically significant, we have not reported in the figure the differences between AuNP and LPS (*p* = 0.0203 for TNFα, and *p* = 0.0009 for IL-6) or between AuNP and LPS + AuNP (*p* = 0.0491 for TNFα, and *p* = 0.0011 for IL-6).

Data in Figure 3 and Figure 4 show the primary and secondary response of monocytes when LPS is used as stimulus. LPS is known as a potent inflammatory activator and as a potent inducer of a tolerance type of memory [8,9]. Monocyte activation was assessed as production of the inflammatory cytokines TNFα and IL-6. As expected, when using endotoxin-free AuNP [30,32], particles did not induce inflammatory cytokine production, while LPS potently induced TNFα and IL-6 production (Figure 3). Co-exposure to LPS and AuNP did not change the reactivity of monocytes to LPS (differences between LPS and LPS + AuNP groups: *p* > 0.05), confirming that AuNP do not directly activate human monocytes, nor do they interfere with LPS-induced inflammatory activation [32]. 

After primary activation, cells were rested in culture for 6 days, during which time they returned to a quiescent status, measured as the return to baseline of inflammatory cytokine production. By visual inspection, cell number in all treated groups was comparable to that in the control wells, with no sign of increased cell death in groups treated with LPS or BCG. Primed quiescent cells were then exposed to culture medium alone or containing a higher LPS concentration (to mimic exposure to a more potent adverse event). Restimulation with medium confirmed that, in all primed cells, cytokine production had returned to baseline, thus achieving a true resting status (Figure 4, the identical lack of response in all primed cells exposed to medium alone is reported as a single Ctrl zero value). Restimulation with LPS induced a significant production of inflammatory cytokines in cells primed with medium alone or with AuNP, indicating that AuNP did not induce any change in the secondary response and are therefore unable to induce memory (as far as these two cytokines are concerned). Confirming previous notions, restimulation of cells previously primed with LPS resulted in significantly reduced production of both TNFα and IL-6 compared to medium-primed cells, indicating the induction by LPS priming of a “tolerance” type of innate memory. Priming with LPS in the presence of AuNP induced a tolerance type of response to the LPS challenge that is indistinguishable from that induced by LPS alone, indicating that AuNP do not interfere with or modulate the LPS-induced innate memory (Figure 4). While this experimental design includes the use of the same stimulus (LPS) for both primary and secondary response, the LPS-induced tolerance memory is non-specific, as similar results can be obtained using different agents at challenge (e.g., zymosan) [33].

### 3.2. Effect of AuNP on Innate Memory Responses Induced by BCG

**Figure 5:**
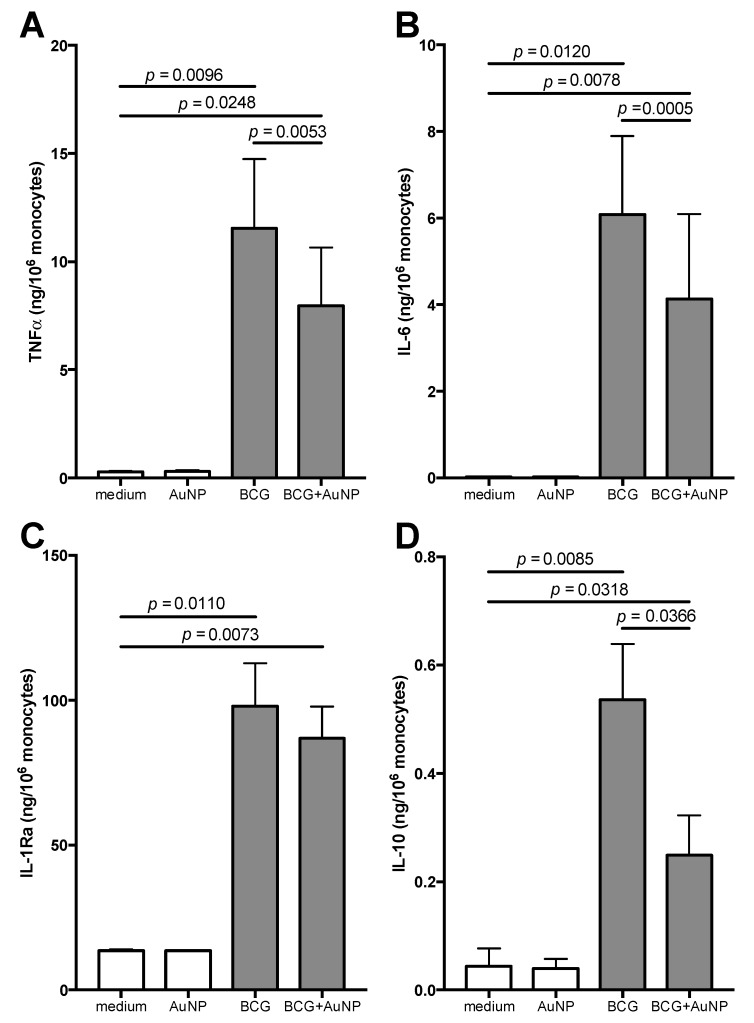
Primary response of human monocytes to BCG and AuNP. CD14^+^ monocytes were exposed for 24 h to BCG (MOI = 1) in the absence or in the presence of AuNP (10 μg/mL). Release of TNFα (**A**), IL-6 (**B**), IL-1Ra (**C**) and IL-10 (**D**) was measured in the supernatants. Results are reported as mean ± SEM of data from 2–4 individual donors tested in separate experiments. *p* values are indicated when *p* < 0.05. Although statistically significant, we have not reported in the figure the differences between AuNP and BCG (*p* = 0.0096 for TNFα, *p* = 0.0120 for IL-6, *p* = 0.0104 for IL-1Ra, and *p* = 0.0099 for IL-10) or between AuNP and BCG + AuNP (*p* = 0.0249 for TNFα, *p* = 0.0078 for IL-6, *p* = 0.0067 for IL-1Ra, and *p* = 0.0498 for IL-10).

We have investigated the effect of AuNP on innate immune response and memory induced by BCG, since this agent has been widely reported as able to induce a potentiated type of innate memory (“trained” immunity) [11,14]. As a priming stimulus in vitro, we have used live BCG, a strain of *Mycobacterium bovis* used as a vaccine against tuberculosis. Exposure to BCG was performed in the same conditions described for LPS except that antibiotics were not added to the culture medium. Thus, freshly isolated cells were exposed for 24 h to medium alone, AuNP, live BCG, or BCG + AuNP, and their activation was assessed as production of the inflammatory cytokines TNFα and IL-6 (Figure 3, upper panels). To better assess the effects of BCG, we have also measured the production of two anti-inflammatory cytokines, i.e., IL-1Ra and IL-10. As already shown in Figure 3, AuNP did not induce monocyte activation in terms of TNFα and IL-6 production (Figure 5, upper panels), and likewise they did not induce production of the anti-inflammatory factors (Figure 5, lower panels). Similar to LPS, stimulation with BCG resulted in a potent monocyte activation, with high production of TNFα and IL-6, and also of IL-1Ra and IL-10. However, at variance with the response to LPS, the presence of AuNP significantly inhibited BCG-induced activation in terms of production of TNFα, IL-6 and IL-10, with a similar trend also for IL-1Ra (Figure 5). 

**Figure 6:**
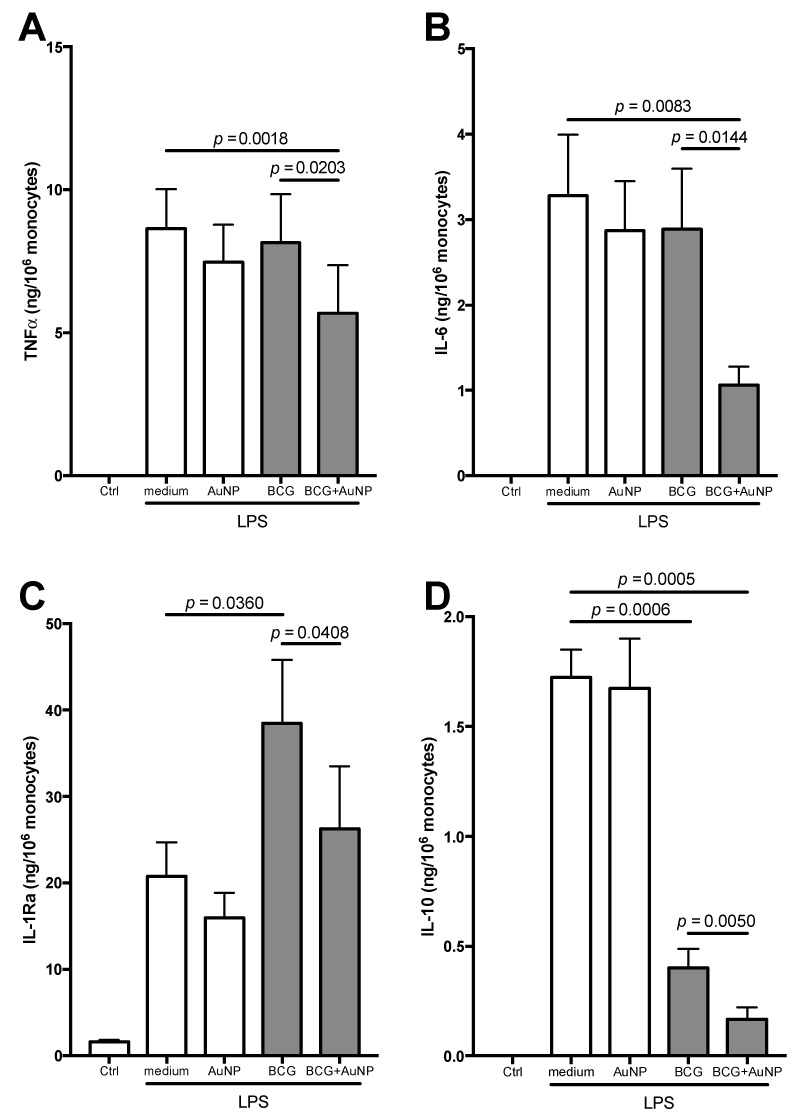
Secondary response of human monocytes primed with BCG and AuNP. Following primary activation (in x-axis: medium, AuNP, BCG, BCG + AuNP), cells were rested for 6 days and then challenged with LPS (5 ng/mL) for 24 h. Production of TNFα (**A**), IL-6 (**B**), IL-1Ra (**C**) and IL-10 (**D**) was measured in the culture supernatants after 24 h. Priming conditions are indicated on the x-axis, and Ctrl represents the response of cells challenged with medium (the response was identical in cells previously primed with medium, AuNP, BCG or BCG + AuNP). Results are reported as mean ± SEM of data from 2–4 individual donors, tested in separate experiments. Further, *p* values are indicated when *p* < 0.05. Although statistically significant, we have not reported in the figure the differences between AuNP and BCG (*p* = 0.0051 for IL-1Ra, and *p* = 0.0145 for IL-10) or between AuNP and BCG + AuNP (*p* = 0.0449 for TNFα, *p* = 0.0056 for IL-6, and *p* = 0.0077 for IL-10).

The induction of innate memory by BCG was assessed with the same in vitro model, by challenging BCG-primed cells with LPS after six days of resting. Although the BCG used for priming was alive, nevertheless after the resting period monocytes had basically returned to a quiescent status, as judged by the background production of inflammatory and anti-inflammatory cytokines (Figure 6). Upon LPS challenge, monocytes previously primed with AuNP produced inflammatory and anti-inflammatory cytokines at the same level as medium-primed cells, confirming that AuNP do not induce innate memory. When BCG-primed cells were challenged with LPS, no memory effect could be seen on the production of the inflammatory cytokines TNFα and IL-6, which was not significantly different from that in medium-primed cells (Figure 6 upper panels). On the other hand, significant and contrasting memory effects were observed in the production of anti-inflammatory factors. The production of the IL-1 inhibitor IL-1Ra in response to a challenge with LPS was twice as high in BCG-primed monocytes compared to medium-primed cells (Figure 6, lower left), while the production of the anti-inflammatory cytokine IL-10 was 4x lower (Figure 6, lower right). 

Cells primed with BCG and AuNP displayed a generalized tolerance type of memory. The production of inflammatory cytokines, not changed by priming with BCG alone or AuNP alone, was significantly reduced in cells primed with BCG + AuNP (Figure 6 upper panels). The production of IL-1Ra, enhanced in BCG primed cells, was back to the levels of medium-primed monocytes in cells primed with BCG + AuNP (Figure 6, lower left). Finally, the strong decrease in IL-10 production in BCG-primed cells was further decreased in monocytes that had been primed with BCG + AuNP (Figure 6, lower right).

## 4. Discussion

The modulation of innate immune memory presents a distinct opportunity toward the prognosis and treatment of inflammation-related disorders and in situations in which a modulation of innate immunity is required (as in the case of vaccine adjuvanticity). The final aim of innate memory is improving the host reaction to subsequent potentially harmful challenges. Such increased fitness can be realized in different ways. A widely described example is the phenomenon known as endotoxin tolerance, which results in a general refractoriness to LPS challenges after a first exposure and aims to prevent extensive tissue damage as consequence of the potent innate immune reaction to LPS [8,9]. Conversely, there is evidence that vaccination with different types of live attenuated vaccines (BCG, *B. pertussis*, *S. typhi*, measles, oral polio vaccine, smallpox) can induce non-specific protection against unrelated pathogens [34,35,36,37,38], a finding that is most likely based on a vaccine-induced innate memory that results in enhanced reactivity, known as “trained” immunity or potentiation [3,11]. On the other hand, some experimental evidence suggests that deranged innate memory may be at the basis of anomalous innate/inflammatory reactions in a number of inflammation-based diseases [39,40]. A fundamental understanding of which agents may induce and modulate innate memory is a critical first step toward a possible future exploitation for medical purposes. In the last 10 years renewed interest in the topic has led to the development of several informative models for studying the non-specific secondary response of innate cells to stimuli such as LPS and BCG [9,11]. The mechanisms underlying the innate memory observed in vivo (tolerance or potentiation) can be studied in vitro using human primary innate cells exposed to a sequence of diverse stimuli. From such studies it is evident that tolerance and potentiation are not an overall decrease or increase in the reactivity of monocytes to stimuli, but rather a re-programming of their responsiveness, with the production of some factors being enhanced while others are produced at a lower level, to reach an overall different response to challenges [33]. While we do not know yet which is the in vitro functional counterpart of a tolerance vs. a potentiation memory, we can hypothesize that, with all due limitations, an LPS-induced memory in human primary monocytes in vitro could represent at least partially the in vivo LPS-induced memory.

In this work we address a new biomedical safety issue, i.e., the possibility that biomaterials used in diagnostic and therapeutic applications could induce an unwanted innate memory or interfere with the induction of a normal (and allegedly protective) innate memory, thereby posing a risk of inadequate protective responses to subsequent challenges. On the other hand, biomaterials that are able to modulate the establishment of memory could be used for inducing protective memory in cases of immune-related diseases in which responses to challenges are excessive (as in autoimmune and chronic inflammatory diseases) or insufficient (as in immunosuppression caused by diseases, cancer, aging). As a model for medically used biomaterials, we have addressed in this study gold nanoparticles, which are used as metal imaging agent and are being developed as drug carriers, due to their high compatibility and lack of toxicity [27]. 

As already observed [32], we have confirmed that AuNP do not induce an innate immune response, ***i.e.***, they are unable to induce monocyte activation *in vitro*. The fact that the AuNP used in this study are endotoxin-free further strengthens the lack of NP activation capacity and underlines the need for an accurate identification of the biologically active contaminants before a reliable assessment of NP toxicity/inflammatory effects can be performed [30,41]. Following some preliminary observations [28], we show in this study that AuNP are also unable to induce an innate memory, assessed as lack of changes in the response to a challenge of cells pre-exposed to AuNP as compared to unprimed cells. In our study, the response to challenge was measured in terms of production of two inflammatory and two anti-inflammatory four cytokines and priming with AuNP does not alter the balance between inflammatory and anti-inflammatory factors produced in response to an infectious/stress challenge (in our case the bacterial molecule LPS). AuNP are known to be readily engulfed by phagocytic cells such as monocytes, and may remain within tissues for an extended duration, yet devoid of cytotoxic effects [42,43,44]. Indeed, our data confirm that AuNP-exposed monocytes are not altered in their reactivity to stimuli. These data add to the evidence for the high tolerability of AuNP and their apparent safety in biomedical application. 

Despite the lack of a direct effect in inducing primary or memory-dependent innate responses, AuNP could still have an effect that needs attention, *i.e.*, they could interfere with the induction of innate memory by microbial agents. We have used in our studies the two best known agents that induce protective tolerance (LPS) and protective potentiation (BCG) in vivo [9,11]. In vitro, LPS-induced tolerance translates into a significant decrease of inflammatory factors such as TNFα and IL-6 in response to a secondary challenge [8,33]. While the secondary challenge can be non-specific, i.e., different from the memory-inducing priming agent (see previous data using zymosan) [33], in this study we have kept LPS also as secondary agent. We have found that priming with LPS in the presence of AuNP neither alters the primary stimulation, nor affects the response of LPS-primed cells to a secondary challenge. Although LPS is only one of a myriad of pattern recognition ligands, from these data we might surmise that if NP do not interfere with primary receptor activation on the extracellular membrane, they do not subsequently alter the cellular pathways of memory establishment.

BCG is a known inducer of the potentiation type of innate memory that, at variance with LPS-induced tolerance, prompts cells both ex vivo and in vitro toward elevated production of inflammatory cytokines such as TNFα and IL-6, and also anti-inflammatory cytokines, in particular IL-1Ra and IL-10 [11,12,14]. We have investigated whether AuNP may modulate the BCG-induced memory, by assessing the production of inflammatory and anti-inflammatory cytokines in vitro in response to LPS as secondary stimulus. Experimentally we used a live *Mycobacterium bovis* BCG of the Moreau strain, which has been (and still is) used as tuberculosis vaccine in Brazil for the past 100 years [45]. Live BCG may enter and remain viable within innate immune cells [46,47]. To make sure that BCG is alive during the memory induction, we have primed cells with BCG in the absence of antibiotics, while antibiotics were present in culture for the resting and challenge phases. As expected, the primary monocyte response to BCG was a significant induction of the inflammatory cytokines TNFα and IL-6, and of the anti-inflammatory factors IL-10 and IL-1Ra. Most interestingly, co-exposure to BCG and AuNP resulted in a significant decrease in the production of TNFα, IL-6 and IL-10, whereas a non-significant tendency to decrease was observed for IL-1Ra. This trend was always evident, with little donor-to-donor variations (data not shown). Since cell viability was not affected, these data indicate that AuNP can down-regulate BCG-induced monocyte activation. Our data do not present evidence of a possible mechanism, but several hypotheses can be formulated. A direct antibacterial effect of AuNP for BCG cannot be excluded, although data supporting this hypothesis are scant [48]. It is also possible that the presence of AuNP could reduce/interfere with the bacterial uptake by monocytes, as shown for *E. coli* uptake by murine macrophage-like tumor cells [49]. However, that the effect of AuNP could be a simple mechanical/steric interference with uptake seems not to be the case, as neither primary response nor memory induced by another particulate agent, zymosan (yeast cells), are affected in the presence of AuNP (Toepfer et al., unpublished). That the presence of AuNP may change the interaction of BCG with cellular receptors (mainly TLR2 and TLR4) is a possibility worth investigating, in light of preliminary data suggesting that AuNP and other metal particles/metals can directly interact with TLR molecules [50,51,52,53].

Upon challenge of BCG-primed cells with LPS, we did not find a memory-induced enhancement of TNFα or IL-6 production, whereas IL-1Ra was increased and the production of IL-10 was strongly decreased. These data are preliminary, as they are the average of results obtained with cells from four individual donors. However, the response in the four donors was impressively similar (so that the data could be averaged). It should be noted that none of the donors was previously vaccinated with BCG or was PPD-positive. At variance with previously published results obtained with the more recent Danish BCG strain [11,34], we have used the Moreau early BCG strain, which is genetically different and with differences in virulence and efficacy [54,55,56]. This may explain some of the discrepancies between our data and previous publications. It should be noted that we have used live BCG at MOI = 1. From our preliminary experience, the memory-inducing capacity of killed BCG is reduced in comparison to live bacteria (unpublished).

The different memory effects observed on different cytokines confirm the hypothesis that innate memory is a global reprogramming of innate immune reactivity, rather than a general decrease or increase of responsiveness. From our data, it seems that BCG-induced memory would attain better protection by maintaining unaltered the capacity to produce inflammatory factors (TNFα and IL-6) but concomitantly decreasing the production of the anti-inflammatory cytokine IL-10, thereby amplifying the innate/inflammatory protective response. Notably, BCG memory results in a significantly higher production of IL-1Ra, which can be interpreted as the need, in a stronger protective response, for a more potent control of IL-1β at the tissue level. The very low production of IL-1β in cells at challenge did not allow us to collect reliable information on the memory-induced modulation of IL-1β production in this study (not shown). 

The finding that AuNP decrease a BCG-stimulated monocyte response led us to predict that the memory induced by priming with BCG + AuNP would be less potent than that induced by BCG alone. Indeed, previous dose-response experiments have shown that the lower the activation in the primary response the less pronounced the induction of memory [33]. However, this was not the case here. BCG priming did not induce changes in the ability of cells to produce TNFα and IL-6 in response to an LPS challenge (no memory induction), while priming with BCG + AuNP significantly decreased the production of inflammatory cytokines, well below the level in unprimed control cells (induction of a tolerance type of memory). The strong increase in IL-1Ra production in BCG-primed cells was abolished in cells primed with BCG + AuNP, which produced IL-1Ra levels comparable to those of unprimed control cells. Eventually, priming with BCG + AuNP induced an even more substantial reduction in the production of IL-10 compared to cells primed with BCG alone. Thus, while BCG priming reprogrammed cell reactivity in a fashion that can be reconducted to better protection at challenge, the presence of AuNP changed the BCG-induced memory towards a generalized tolerance. If this is confirmed in further studies with different microbial priming agents, we may consider the possibility of using AuNP for reducing excessive innate/inflammatory reactivity in inflammatory, degenerative and autoimmune diseases.

The inhibitory effect of AuNP on BCG-induced memory may be only partially explained by their behavior in the primary response, since priming with BCG with AuNP could decrease the response at challenge even in cases in which BCG alone had no effect (production of TNFα and IL-6), thus the AuNP effect could not be fully attributed to a reduction in the priming efficacy of BCG. We may hypothesize that the presence of AuNP changes the uptake and intracellular fate of BCG, thereby affecting not only the primary response to BCG, but also the BCG-dependent epigenetic or metabolic mechanisms of innate memory induction. It is known that innate immune memory depends on epigenetic changes that predispose cells toward different (increased or decreased) transcription and translation of target genes upon restimulation, mostly based upon modification of histone proteins [2,57]. Innate memory is also dependent upon the cell metabolic status, with elevated baseline production of metabolites such as lactate being indicative of increased inflammatory reactivity (e.g., production of TNFα and IL-6) [58,59,60]. We may speculate that AuNP interfere with the intracellular processes that govern histone modifications in response to BCG and/or with metabolic processes induced BCG, and in turn affecting the subsequent cell reactivity to restimulation. 

## 5. Conclusions

Innate immune memory is critical to maintaining successful host defense responses to multiple challenges. In this study, we show that AuNP can interfere with the induction of memory by live bacteria by producing a generalized tolerance type of memory. This resulted in significantly lower production of inflammation-related factors upon challenge. AuNP are non-toxic biocompatible particles that have been used since the early 1950s for tumor therapy (as radioactive colloidal gold in prostate cancer) and are currently being developed for several therapeutic strategies. Apart from their inability to directly trigger immune reactions (which makes us consider them as non-immunogenic and immunologically safe/inert), our study shows that AuNP are equally unable to directly induce innate memory in human primary monocytes. The key finding that AuNP can however modulate the innate memory induced by live bacteria presents a promising scenario in view of future approaches to immunotherapy, in particular for diseases in which excessive immune reactions constitute the basis of the pathology (autoimmunity, chronic inflammatory diseases, degenerative, and neurodegenerative diseases, cytokine storm in severe infections, etc.). A further consideration is that, when assessing safety of NP or any compound, the absence of direct immune-related effects may not provide sufficient proof of lack of effect, and that “real-life” situations, such as the concomitant presence of microorganisms, must be explored.

## Supplementary Materials

The following are available online at https://www.mdpi.com/2073-4409/9/2/284/s1.
