# Supplementary material for: Gold Nanoparticles Modulate BCG-Induced Innate Immune Memory in Human Monocytes by Shifting the Memory Response towards Tolerance
Source: Cells. 2020 Jan 23;9(2):284. doi: 10.3390/cells9020284 (PMC7072314; doi:10.3390/cells9020284)
Supplement: Supplementary file 1 [file cells-09-00284-s001.pdf]

cells

MDPI

SUPPLEMENTARY MATERIAL

Gold nanoparticles modulate BCG-induced innate immune memory in human monocytes by shifting the memory response towards tolerance

Swartzwelter at al.

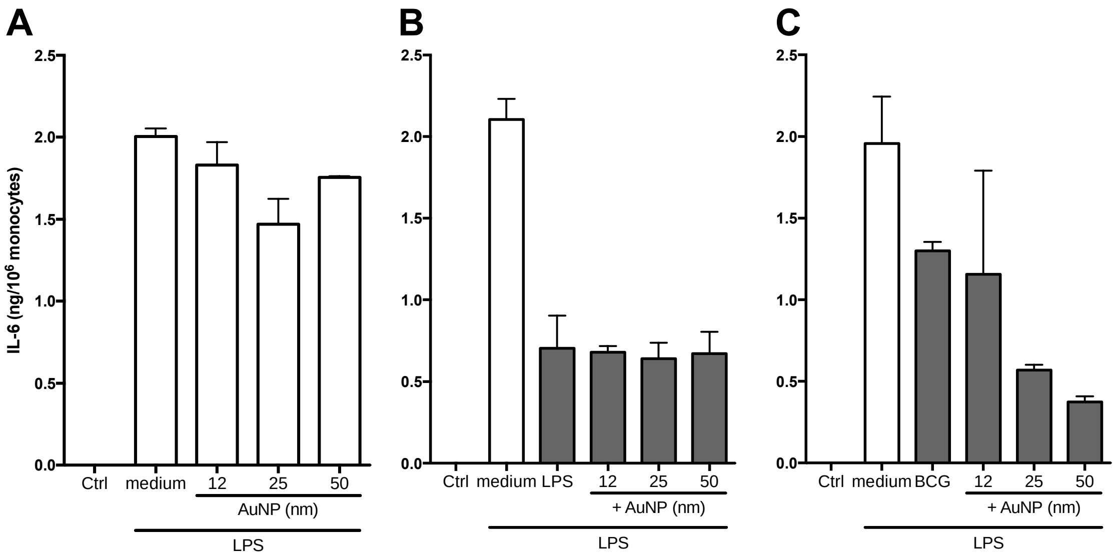

Supplementary Figure S1. Size-dependent effect of AuNP on stimulus-induced memory.

Primary monocyte activation was performed for 24 h with medium alone (A), LPS 1 ng/ml (B) or BCG MOI=1 (C) in the absence or in the presence of AuNP of different diameter (12.5, 25, 50 nm). AuNP were used at the same concentration in terms of surface area (127 mm^2/ml), corresponding to 4.8, 10 and 20 μg/ml, respectively. Cells were then rested for 6 days and re-challenged with LPS (5 ng/ml) for 24 h. Supernatants were tested for IL-6. Ctrl represents the response of cells challenged with medium (the lack of response was identical in cells previously primed with medium or any other stimuli and combinations). Results are reported as mean ng IL-6/10^6 cells ± SD of biological replicates from 1 individual donor, representative of 2 tested.

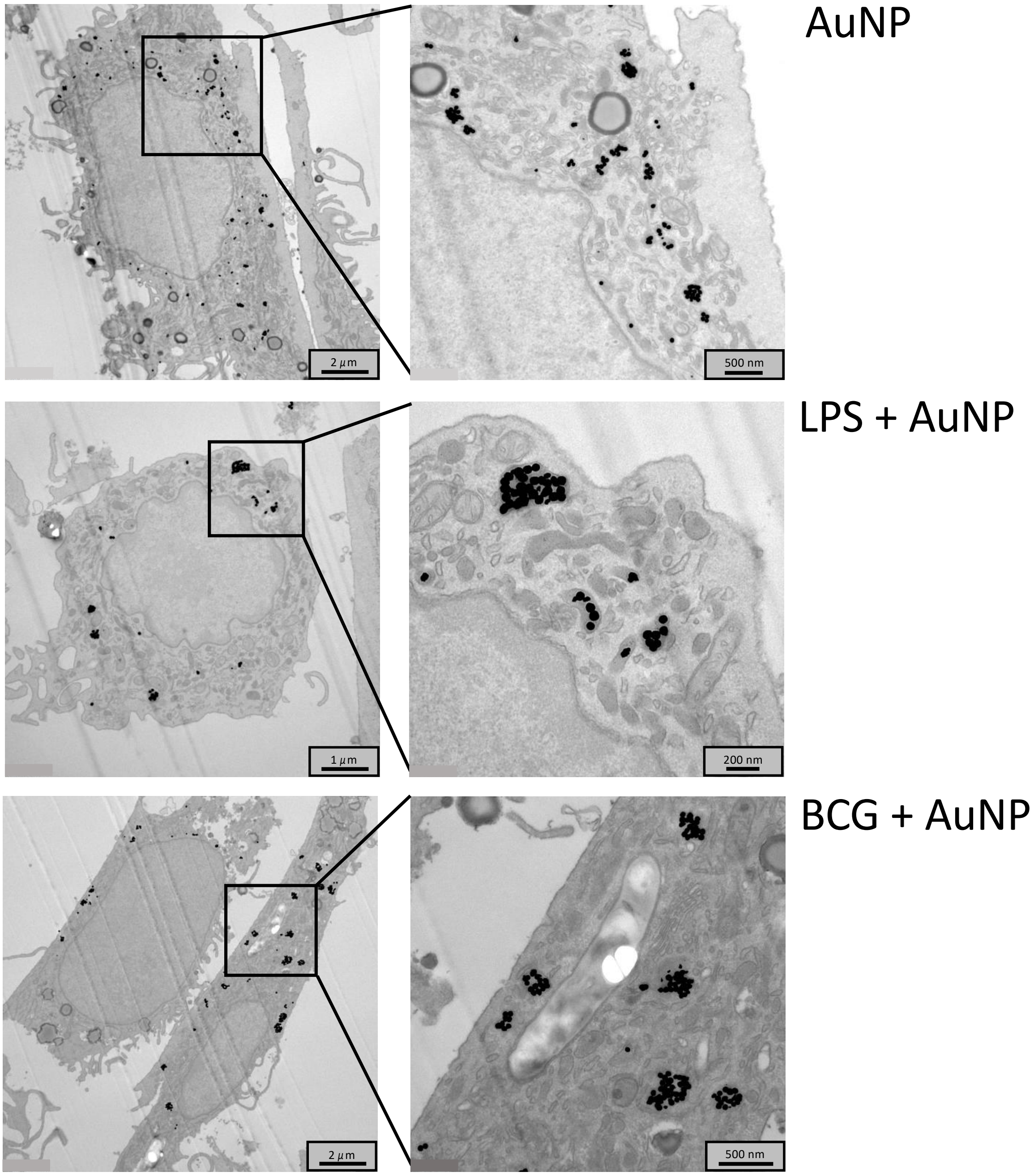

Supplementary Figure S2. TEM analysis of AuNP uptake.
Cells were exposed for 24 h to AuNP alone (upper panels) or in the presence of LPS (center panels) or BCG (lower panels). NP uptake was examined by transmission electron microscopy.
